# Percutaneous Bilateral Endoscopic Lumbar Interbody Fusion: Technical Note and Preliminary Results

**DOI:** 10.1155/2022/2227679

**Published:** 2022-04-11

**Authors:** Huan Chen, Huan Zhang, Erping Yang, Qinjie Ling, Erxing He

**Affiliations:** ^1^Spine Surgery, The First Affiliated Hospital of Guangzhou Medical University, Guangzhou, China; ^2^Department of Orthopedics, Huanggang Central Hospital of Yangtze University, Hubei, China; ^3^Huanggang Central Hospital of Yangtze University, Hubei, China

## Abstract

**Objective:**

The purpose of this study was to investigate the feasibility and clinical efficacy of the percutaneous bilateral endoscopy technique (microendoscopic trans-Kambin's triangle lumbar interbody fusion + percutaneous endoscopic transforaminal decompression of the lumbar spinal canal, ME-TKT-LIF+ PETD) in the treatment of lumbar degenerative diseases.

**Methods:**

From May 2016 to September 2018, 29 patients (16 males and 13 females) who suffered from neurologic symptoms due to degenerative lumbar spine disease and underwent percutaneous bilateral endoscopy technique were enrolled. A microendoscope was used for fusion, and a percutaneous endoscope was used for spinal canal decompression. These patients' perioperative and clinical outcome-related parameters were collected and analyzed.

**Results:**

The mean intraoperative blood loss was 72.8 ± 40.6 ml, the operation time was 87.1 ± 10.1 min, the postoperative ambulatory time was 1.69 ± 1.0 days, the hospital stay was 2.6 ± 1.3 days, and the follow-up period was 22.34 ± 4.2 months. The visual analog scale (VAS) and the Oswestry disability index (ODI) were significantly improved at the early postoperative and last follow-up, respectively. According to the modified MacNab criteria, 11 (11/29) cases were rated as excellent, 15 (15/29) as good, and 3 (3/29) as fair, and the excellent and good rate was 89.7%. Twenty-eight (28/29) cases demonstrated solid fusion, and the fusion rate was 96.6%.

**Conclusion:**

The percutaneous bilateral endoscopy technique is safe and feasible in the treatment of lumbar degenerative diseases, with the advantage that more normal anatomical structures are preserved. It is an optional method of lumbar interbody fusion.

## 1. Introduction

In 1997, Foley first carried out microendoscopic discectomy (MED) to treat lumbar degenerative diseases. Paravertebral muscles were bluntly dilated by cannulas, and the operation was performed under microendoscopy [1]. The idea of minimally invasive surgery and endoscopic technology has indeed been introduced into spinal surgery. In 2006, Ruetten et al. developed percutaneous endoscopic lumbar discectomy (PELD) [2]. PELD causes little damage to the normal anatomical structure, and its clinical efficacy has been widely verified [3].

Based on the surgical techniques described above, various lumbar interbody fusion procedures, such as microendoscopic transforaminal lumbar interbody fusion (ME-TLIF), percutaneous endoscopic transforaminal lumbar interbody fusion (PE-TLIF), biportal endoscopic transforaminal lumbar interbody fusion (BE-TLIF), and endoscopic lumbar interbody fusion (Endo-LIF), have recently been performed [4–8]. Overall, endoscopic approaches to these procedures can be divided into two types: posterolateral and trans-Kambin's triangle approaches. The posterolateral approach was similar to traditional minimally invasive surgery transforaminal lumbar interbody fusion (MIS-TLIF), with the articular processes and a part of the vertebral lamina still needing to be excised [9, 10]. Although the trans-Kambin's triangle approach was less invasive than the posterolateral approach, it had limited decompression of the central spinal canal and the traversing nerve root [11]. To preserve normal anatomical structures such as the lamina and articular processes and allow the nerve tissues in the spinal canal to be directly decompressed, we performed the percutaneous bilateral endoscope technique. This technique included microendoscopic trans-Kambin's triangle lumbar interbody fusion (ME-TKT-LIF) and percutaneous endoscopic transforaminal decompression of the lumbar spinal canal (PETD). A microendoscope was used for fusion, and a percutaneous endoscope was used for spinal canal decompression. The purpose of this retrospective study was to investigate the feasibility and clinical efficacy of the percutaneous bilateral endoscopy technique (ME-TKT-LIF+ PETD) in the treatment of lumbar degenerative diseases.

## 2. Material and Methods

### 2.1. Patient Data

This retrospective study included 29 patients who all presented with neuropathic intermittent claudication and radicular leg pain (May 2016 to September 2018). The inclusion criteria were as follows: (1) lumbar degenerative/isthmic spondylolisthesis grade 1 or 2; (2) lumbar disc herniation with segmental instability; and (3) lumbar foraminal stenosis with segmental instability. Patients were excluded as follows: (1) lumbar spondylolisthesis grade 3 or above; (2) severe lumbar canal stenosis; (3) L5-S1 segment lesion with a high iliac crest; and (4) tumors, infections, and fractures involving lumbar vertebrae.

A total of 16 males and 13 females were recruited in this study, with an average age of 59.4 ± 9.1 years (range 42 to 74). Seventeen patients were diagnosed with lumbar spondylolisthesis, 9 with lumbar disc herniation with segmental instability, and 3 with lumbar foraminal stenosis with segmental instability. All patients had single-segment lesions: L3-L4 in 3 patients, L4-L5 in 22 patients, and L5-S1 in 4 patients (Table 1). Preoperative symptoms in all patients were unilateral radicular radiation pain with or without intermittent claudication.

### 2.2. Surgical Technique

**Figure 1:**
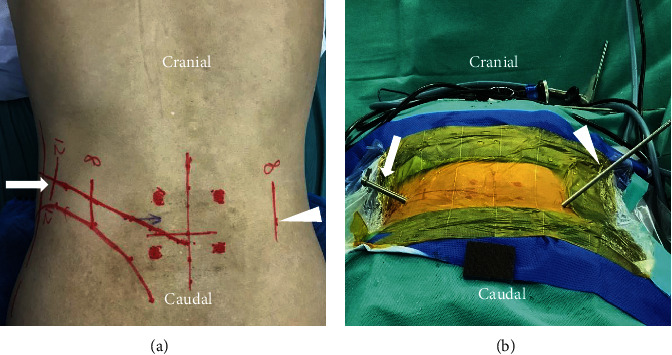
Mark line on skin, the incisions of PETD and ME-TKT-LIF are, respectively, represented by the arrow and triangle (a). The working channel of the percutaneous endoscope (arrow), the primary guide rod (triangle) (b).

The procedures of all our patients were performed under general anesthesia and took the prone position on the operating table when C-arm fluoroscopy was feasible. Somatosensory-evoked potentials, motor-evoked potentials, and electromyography were used to monitor the involved nerve roots. Cushions were placed on the chest and hip joints, leaving the abdomen hanging. According to the standard anteroposterior position, the direction and angle line of the intervertebral foramen approach on both sides of the lesion segment were determined under C-arm fluoroscopy and marked on the skin (Figure 1). The surgical site of the patient was routinely sterilized and covered with sterile and waterproof towels (taking the L4/5 segment as an example).

**Figure 2:**
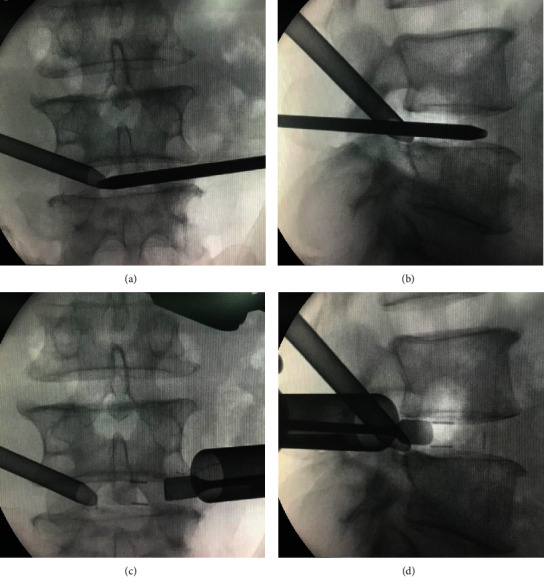
Intraoperative fluoroscopy. Working channel of PETD and primary guide rod of ME-TKT-LIF (a, b). Two working channel, exiting nerve root retractor, and cage (c, d).

**Figure 3:**
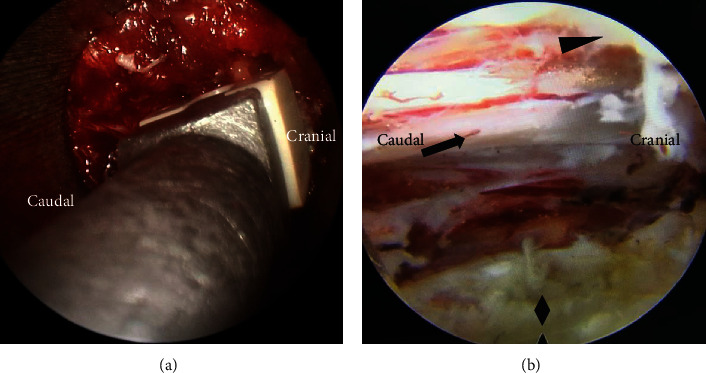
Intraoperative images of ME-TKT-LIF, cage was implanted under microscopic endoscope (a). Intraoperative images of PETD, ligamentum flavum (triangle), traversing nerve root (arrow), intervertebral disc (rhombus) (b).

Percutaneous endoscopic transforaminal decompression of the lumbar spinal canal (PETD) (Figure 1): On the symptomatic side of the patient, a primary guide rod with a diameter of approximately 4 mm and a blunt tip was inserted at a distance of approximately 10 to 14 cm from the midline of the vertebral column, with the direction pointing to the superior articular process. After the tip of the primary guide rod touched the superior articular process, C-arm fluoroscopy was performed to confirm contact. The guide rod was slid to the ventral side and advanced 1-2 cm to the intervertebral disc through the intervertebral foramen, which was again confirmed by C-arm fluoroscopy. The surgical incision on the skin was extended to a length of 7 mm. The soft tissue was gradually expanded through a series of retractors. A part of the superior articular process was excised by the trepan under the cannula with a hook at the end, which can prevent the nerve root and epidural sac from being damaged. The location reached by the terminal of the trepan was confirmed by fluoroscopy, if necessary. The trepan and the cannula were replaced by the 6.9-mm beveled working channel (Figure 2), and then, the endoscope was placed. On the basis of the patient's preoperative symptoms, physical signs, and imaging results, the degenerated nucleus pulposus tissue was removed, and the spinal canal was decompressed accurately under continuous irrigation with normal saline (Figure 3).

**Figure 4:**
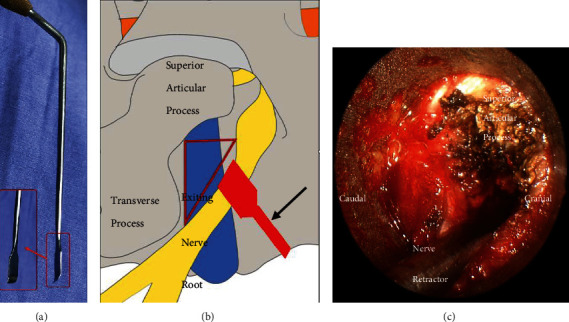
Special nerve retractor (a). Anatomy graph that showed the special nerve retractor dilating the exiting nerve root to enlarge the Kambin's Triangle. Red triangle line was the Kambin's triangle. The black arrow pointed to the special nerve retractor (b). Surgical vision of working place under the ME-TKT-LIF system. The superior articular process was partially resected under endoscope (c).

Microendoscopic trans-Kambin's triangle lumbar interbody fusion (ME-TKT-LIF) (Figure 1): On the opposite side, as in the abovementioned surgical method, a 4 mm primary guide rod directed to the superior articular process was inserted at a distance of approximately 6 to 8 cm from the midline, and the blunt tip of the guide rod reached the surface of the superior articular process under the control of C-arm fluoroscopy (Figure 2). After that, the tip of the guide rod was slid to the ventral side and then entered the center of the intervertebral space through the intervertebral foramen at an angle of approximately 40-50 degrees; its depth and final position were adjusted through C-arm fluoroscopy. Through the skin incision extended to 2.5 cm, soft tissue progressive dilatation was performed. Except for the primary guide rod, the dilatation cannula and working channel were not inserted into Kambin's triangle but instead just placed on the outer surface of the superior articular process. Then, the dilatation cannula was removed, and only the primary guide rod and working channel were retained. After the microscopic endoscope system was fixed on the working channel with a diameter of 22 mm, the primary guide rod was removed under full visibility, and the exiting nerve root was protected by a special nerve retractor (Figure 2). “Special nerve retractor” has been approved by the ethics committee of author's hospital before clinical application. Under the endoscope, the special nerve retractor was used to dilate the exiting nerve root to the ventral side moderately, which enlarged the Kambin's triangle, and played a barrier role to protect the exiting nerve root. The superior articular process was then partially excised, and the Kambin's triangle was further enlarged (Figure 4). In some patients with foraminal stenosis, we performed foraminoplasty under microendoscopic visualization. We used reamers, curettes, and forceps for endplate preparation, and most of the annulus fibrosus and nucleus materials in the intervertebral space were removed to reach the cranial and caudal endplates. The raspatories were used to determine the size of the cage. Under the fine visualization of the endoscope, the mixed bone chips of autogenous bone and allograft bone were implanted into the intervertebral space, and then, the cage with the optimal size corresponding to the height of the intervertebral space was gently hammered into the intervertebral space (Figure 3). The size of the cage was equivalent to that used in conventional open surgery, and finally, the integrity check of the exiting nerve root was performed. Nerve structures in the spinal canal need not be exposed.

**Figure 5:**
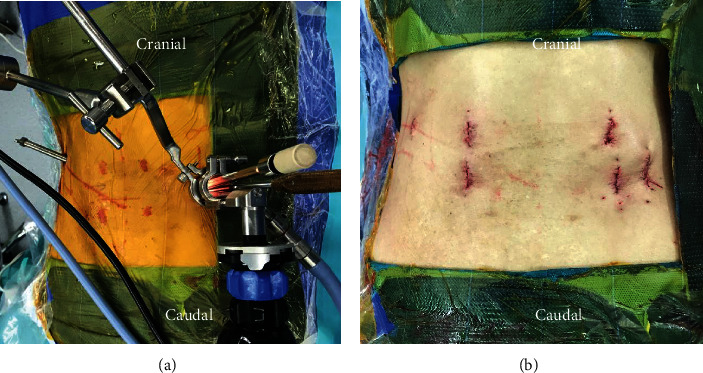
Panoramic photograph of the percutaneous bilateral endoscope technology (a). The photo shows the stitched incision (b).

**Figure 6:**
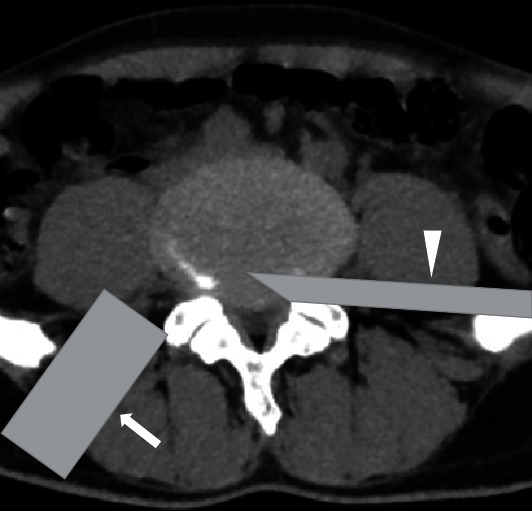
Pattern diagram of the location of the two working channels during the operation, PETD working channel (triangle) on the symptomatic side, ME-TKT-LIF working channel (arrow) on the opposite side.

**Figure 7:**
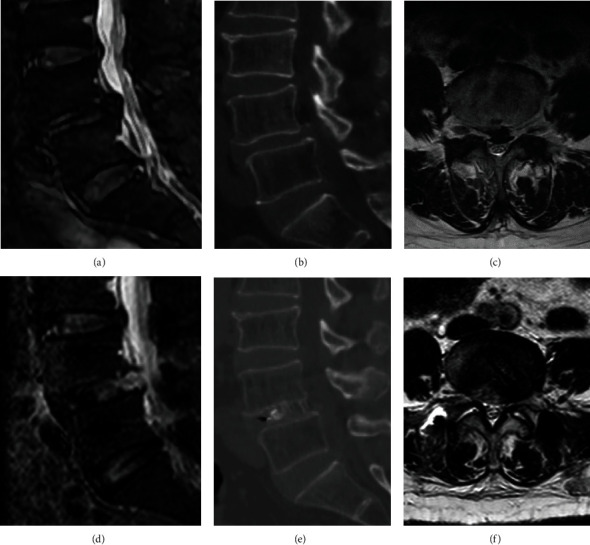
Case examples with pre- and postoperative magnetic resonance imaging (MRI) scans and computed tomography (CT) scans. A 73-year-old female patient was diagnosed as lumbar spondylolisthesis before operation. Preoperative sagittal MRI scan (a), preoperative sagittal CT scan (b), preoperative transverse MRI scan (c), postoperative sagittal MRI scan (d), postoperative sagittal CT scan (e), and postoperative transverse MRI scan (f).

Percutaneous pedicle screws and connecting rods were implanted without placing drainage tubes, and then, the incision was sutured intradermally (Figures 5–7).

### 2.3. Clinical Assessment

The following parameters were recorded: operation time; blood loss; postoperative ambulatory time; hospitalization time; follow-up time and complications; and VAS and ODI scores for lumbar and lower extremity pain preoperatively, 1 month postoperatively, and 1 year postoperatively. The satisfaction of clinical outcomes at the last follow-up was assessed using modified MacNab criteria and was divided into four grades: excellent, good, fair, and poor. Flexion-extension lateral radiography and computed tomography were used to evaluate intervertebral fusion [12]. The clinical efficacy was assessed by an independent physician who was blinded to all patients.

### 2.4. Statistical Analysis

VAS and ODI scores before and after operation were compared by a paired-sample Wilcoxon signed rank test. In this study, SPSS version 17.0 (SPSS, Chicago, IL, USA) was used for statistical analysis, and *P* < 0.05 was set as the level of significance.

## 3. Results

This study included 29 patients (16 males and 13 females) with lumbar degenerative diseases, with a mean age of 59.4 ± 9.1 years (range 42-74 years), operation time of 87.1 ± 10.1 min (range 65-110 min), intraoperative blood loss of 72.8 ± 40.6 ml (range 35 ml-250 ml), postoperative ambulatory time of 1.69 ± 1.0 days (range 1-5 days), hospitalization time of 2.6 ± 1.3 days (range 1-7 days), and follow-up period of 22.34 ± 4.2 months (range 12-24 months). The VAS for preoperative leg pain was 7.5 ± 0.7, the VAS for preoperative back pain was 4.4 ± 1.3, the VAS for leg pain 1 month postoperation was 1.3 ± 0.8, the VAS for back pain 1 month postoperation was 1.7 ± 0.9, the VAS for leg pain 12 months postoperation was 0.9 ± 0.7, the VAS for back pain 12 months postoperation was 0.5 ± 0.6, the preoperative ODI (%) was 61.0 ± 4.6, the ODI (%) 1 month postoperation was 19.5 ± 4.0, and the ODI (%) 12 months postoperation was 17.6 ± 2.4. The VAS and ODI scores before and after the operation were significantly different (*P* ≤ 0.001). At the last follow-up, 28 (28/29) cases demonstrated solid fusion through computed tomography and flexion-extension lateral radiography, and the fusion rate was 96.6%. According to the modified MacNab criteria, 11 (11/29) cases were rated as excellent, 15 (15/29) as good, and 3 (3/29) as fair, and the excellent and good rate was 89.7% (Table 2).

## 4. Discussion

For lumbar degenerative diseases requiring interbody fusion, various procedures such as MIS-TLIF, ME-TLTF, PE-TLIF, BE-TLIF, and End-LIF have been developed at present [4–8]. These techniques are superior to traditional open surgery in trauma, bleeding volume, and postoperative rehabilitation [13]. As described in the literature, surgical approaches can be divided into posterolateral approaches and trans-Kambin's triangle approaches. The advantage of the posterolateral approach is that the central spinal canal and the traversing nerve roots can be effectively decompressed but require excessive resection of normal anatomical structures, such as the articular process joint, laminae, ligamentum flavum, and epidural fat. After these normal tissues are excised, obvious epidural scars will form postoperatively [14, 15]. In contrast to the posterolateral approach, the trans-Kambin's triangle approach required only the resection of approximately 1/4 of the superior articular process while preserving most of the normal anatomy. However, this approach can only decompress the exiting nerve root, with limited decompression for the traversing root and spinal canal [16, 17]. The efficacy of PELD for spinal canal decompression has been demonstrated [10]. The bilateral endoscope technology introduced in this paper combines trans-Kambin's triangle approach and PELD to complement each other's shortcomings.

ME-TKT-LIF was developed on the basis of MED. ME-TKT-LIF is an air-based endoscopic lumbar fusion procedure, and the operating instruments are similar to those of traditional open surgery. However, the surgery was performed under the channel and endoscope, and the learning curve was steep. The primary guide rod of ME-TKT-LIF is passed through Kambin's triangle into the intervertebral space at an angle of approximately 45°, similar to the YESS technique [18]. The working channel is placed at the external orifice of the intervertebral foramen, with the superior articular process as the fulcrum. The dural sac and the traversing nerve root are not exposed, and the exiting nerve root is protected by the nerve root retractor under visualization. The height and width of the lumbar Kambin's triangle are 12-18 mm and 10-12 mm, respectively [19]. According to the description of Andrew [20], Kambin's triangle is a Mitsubishi cone with a small outer part and a large inner part, so only approximately 1/4 of the superior articular process needs to be removed to complete the implantation of the cage. ME-TKT-LIF uses a large 22-mm channel with extensive and reasonable removal of the annulus fibrosus and the cartilage endplate under full endoscopy. The unrestricted choice of cage size and type, along with adequate interbody bone grafting, is prerequisite for improved fusion rates. The articular process, ligamentum flavum, posterior longitudinal ligament, and epidural fat were not removed, thus reducing the possibility of epidural scar formation and shortening the operation time. The complications of PE-TLIF have been reported in the literature to be 20% to 35%, mainly including endplate collapse, fusion failure, and pedicle screw or rod fracture [21]. Only expandable cages or small cages can be used in PE-TLIF due to the working channel diameter of 10–12 mm.

The ME-TKT-LIF procedure is an indirect decompression process for the central spinal canal. Obviously, in patients with symptoms of radioneuralgia, indirect decompression alone is not feasible. However, the most obvious advantage of PETD is the direct and accurate decompression of the spinal canal while preserving normal anatomical structures such as the articular process joint, ligamentum flavum, and epidural fat. The establishment of the working channel of ME-TKT-LIF and PETD is performed simultaneously on different side, which can reduce the numbers of fluoroscopic examination and shorten the operation time. In this technique, the establishment of the working channels of ME-TKT-LIF and PETD all depends on the integrity of the superior articular process. PETD and ME-TKT-LIF both need to use the primary guide rod first. The primary guide rod, approximately 4 mm in diameter and blunt in tip, is inserted with its direction pointing to the superior articular process. After the tip of the guide rod reach the superior articular process, the guide rod is slid to the ventral side and into the intervertebral disc through the intervertebral foramen. If the two processes performed on the same side, after the PETD is finished, the superior articular process is incomplete. In ME-TKT-LIF, the primary guide rod cannot utilize this anatomy; it is hard to establish a working channel and the risk of nerve root increase. The operation was performed under general anesthesia, and the peripheral nerves involved were monitored using somatosensory-evoked potentials, motor-evoked potentials, and electromyography, which improved patient comfort and ensured safety. The primary guide with a diameter of 4 mm and a bullet-shaped tip also help to avoid injury to the exiting nerve roots.

The mean operative time in this study was 87.1 ± 10.1 min. During the operation, it is not necessary to resect the articular process joint, and the simultaneous placement of the working channel on both sides can help to shorten the operation time. The mean intraoperative bleeding volume was 72.8 ± 40.6 ml. The mean postoperative ambulation time was 1.69 ± 1.0 days, and the mean hospital stay was 2.6 ± 1.3 days. Similar to other minimally invasive endoscopic lumbar fusion techniques, this technique outperforms MIS-TLIF in terms of bleeding volume, postoperative ambulation time, and hospital stay. The mean time to ambulation and discharge after MIS-TLIF is up to 3.2 days and 9.3 days, respectively [22]. For MIS-TLIF, most surgeons used a retractor in combination with a microscope [23], while we used a series of expanding cannulae and a working channel. ME-TKT-LIF is an endoscopic TLIF, not a microscope-assisted TLIF. The retractor is more traumatic to the muscle than to the channel [24]. ME-TKT-LIF utilizes the natural structure of the intervertebral foramen, and the angle and direction of its working channel can be easily adjusted without removing the zygapophyseal joint, thus completing the endplate preparation well. However, MIS-TLIF requires the resection of more normal anatomical structures to complete the decompression of the target area. In the present study, the postoperative VAS score (back pain and leg pain) and ODI both improved significantly compared to presurgery scores. Preoperatively, the patient presented with intermittent claudication and radicular pain in the lower extremities, and the mean VAS score for leg pain was higher than the mean VAS score for back pain. The decompression of the dural sac and nerve root by PETD ensures satisfactory short-term postoperative efficacy. This study showed that the fusion rate and efficacy satisfaction of this technique reached 96.6% and 89.7%, respectively. The use of a large 22-mm channel can both adequately and effectively resect the diseased intervertebral disc and implant an intervertebral cage of the same size as that for open surgery to ensure osseous fusion. Previous studies have shown that lumbar intervertebral fusion employs a stand-alone endoscopic TLIF procedure with a complication rate of up to 36% [25]. Therefore, fusion should be accompanied by percutaneous pedicle screws or others internal fixation. ME-TKT-LIF and percutaneous pedicle screws provide the assurance of satisfactory rates of long-term therapeutic results.

In one case, the symptoms of lower extremity pain were not resolved significantly after surgery, and MRI examination revealed partial residue in the spinal canal. After conservative treatment, the VAS score at 1 month was 2. Intraoperative incomplete decompression was considered. At the last follow-up, computed tomography of one patient showed no obvious trabecular bone bridge in the intervertebral space, and the patient had no symptoms. Measures to continue follow-up observations were given.

This research and this technology have deficiencies. First, the sample size of this study was small, there was an absence of a control group, and the mean follow-up period was not long enough, which may not be sufficient to prove the effectiveness of this procedure. Second, surgeons need to be proficient in both endoscopic techniques, and the learning curve is steep. Third, ME-TKT-LIF and PETD are mechanical combinations in this technology, and a better model needs to be developed.

## 5. Conclusion

Under appropriate patient selection and surgical indications, the percutaneous bilateral endoscopy technique is effective and feasible for the treatment of lumbar degenerative diseases, with the advantage that more normal anatomical structures are preserved. It is an optional method of lumbar interbody fusion.

## Figures and Tables

**Table 1:** Demographic characteristics of patients.

Characteristics	Values
Mean age(years)	59.4 ± 9.1
Sex (M/F)	16/13
Diagnosis	
Lumbar spondylolisthesis	17
Lumbar disc herniation with segmental instability	9
Lumbar foraminal stenosis with segmental instability	3
Level of fusion	
L3-L4	3
L4-L5	22
L5-S1	4

Note: mean values are presented as mean ± standard deviation; M = Male, F=Female.

**Table 2:** characteristics of clinical outcome.

Characteristics	Values
Follow-up period	22.34 ± 4.2
Blood loss	72.8 ± 40.6 ml
Operation time	87.1 ± 10.1 min
Postoperative ambulatory time	1.69 ± 1.0 days
Hospitalization time	2.6 ± 1.3 days
VAS for leg pain	
Preoperative	7.5 ± 0.7
1 month postoperative	1.3 ± 0.8 (*P* ≤ 0.001)
12 months postoperative	0.9 ± 0.7 (P ≤ 0.001)
VAS for back pain	
Preoperative	4.4 ± 1.3
1 month postoperative	1.7 ± 0.9 (P ≤ 0.001)
12 months postoperative	0.5 ± 0.6 (*P* ≤ 0.001)
ODI (%)	
Preoperative	61.0 ± 4.6
1 month postoperative	23.1 ± 6.6 (*P* ≤ 0.001)
12 months postoperative	20.1 ± 5.1 (P ≤ 0.001)
MacNab	
Excellent	11(11/29)
Good	15(15/29)
Fair	3(3/29)
Poor	0
Excellent and good rate	89.7%

Note: mean values are presented as mean ± standard deviation; *P* ≤ 0.001 versus preoperative data.

## Data Availability

The data used to support the findings of this study are available from the corresponding author upon request.
